# Nanoparticle-Enabled Interfacial Kinetic Control of Bond-Exchange Reactions: Insights from Biomedical Poly(ε-caprolactone)-Based Dynamic Polymers

**DOI:** 10.1021/acsmacrolett.6c00292

**Published:** 2026-08-10

**Authors:** Klementina Pušnik Črešnar, Tadej Slatinek, Lidija Fras Zemljič, Sandra Schlögl

**Affiliations:** † Faculty of Mechanical Engineering, University of Maribor, SI-2000 Maribor, Slovenia; ‡ Faculty of Chemistry and Chemical Engineering, University of Maribor, SI-2000 Maribor, Slovenia; § 226178 Polymer Competence Center Leoben GmbH, AT-8700 Leoben, Austria; ∥ Institute of Chemistry of Polymeric Materials, Technical University of Leoben, AT-8700 Leoben, Austria

## Abstract

Poly­(ε-caprolactone) (PCL)-based dynamic polymers are promising adaptive biomaterials due to their biocompatibility, biodegradability, and high chain mobility; however, the onset temperature for bond-exchange reactions, often referred to as topology-freezing temperature, *T*_v_, remains difficult to control and is typically treated as a bulk property governed by catalyst selection and network structure. Consequently, many reported dynamic polymers operate at temperatures well above the biomedical operating window, limiting their biomedical applicability. Here, we propose an interfacial kinetic engineering strategy in which nanoparticle–polymer interfaces act as localized kinetic regulators of dynamic covalent bond exchange. By tailoring nanoparticle (NP) surface chemistry, including functional group density, polarity, and catalytic activity, the activation energy (*E*_a_) can be modulated at the nanoscale, enabling programmable control of *T*_v_. Exchange kinetics in PCL-based dynamic polymers are expected to be highly sensitive to the local chemical environment, indicating that interfacial effects can decouple dynamic behavior from bulk composition. This perspective establishes NP–dynamic polymer interfaces as a programmable kinetic design parameter for controlling bond-exchange reactions in dynamic polymer networks, opening a pathway toward spatially defined covalent adaptable materials with application-specific and programmable dynamic behavior.

## Graphical abstract

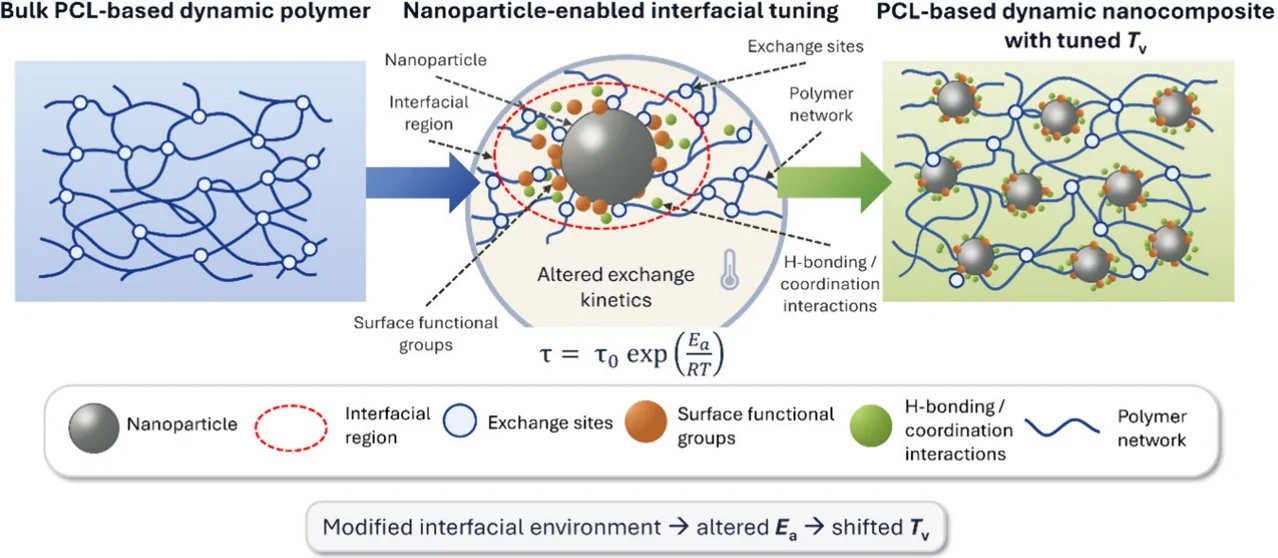

## 1 Introduction

The onset temperature of bond-exchange reactions in dynamic polymers is commonly described by the topology-freezing temperature (*T*_v_), where the subscript “v” denotes “vitrimer.” *T*_v_ strongly influences reprocessability, stress relaxation, and creep resistance. Although often treated as an intrinsic material constant, *T*_v_ is in fact an emergent kinetic parameter, governed by the activation energy (*E*_a_), molecular mobility, and the experimental time scale used for its determination. As a result, reported values vary widely, limiting predictive design and hindering comparison across dynamic polymers.^1–7^ Precise control of *T*_v_ is therefore critical for application performance, particularly in biomedical materials, where dynamic rearrangement must be suppressed under physiological baseline conditions yet activated under externally applied mild-to-moderate thermal stimuli within biomedical operating windows, typically around 45–70 °C.^8–10^ Achieving this balance remains a fundamental challenge for currently available dynamic polymers.

Poly­(ε-caprolactone) (PCL) represents a highly relevant material platform for biomedical dynamic polymers due to its biodegradability, biocompatibility, and intrinsically high bulk segmental mobility, as reflected in its low glass transition temperature (*T*_g_ ≈ −60 °C). While this mobility facilitates dynamic bond exchange, it also complicates precise control of network rearrangement, as even moderate reductions in *E*_a_ can significantly shift the dynamic operating window. PCL-based dynamic polymers, most commonly relying on transesterification reactions, exhibit tunable stress relaxation, reprocessability, and shape-memory behavior.^1–3,11–15^ However, their dynamic response remains governed primarily by global network parameters such as catalyst type and concentration, limiting the ability to directly program *T*_v_.

This limitation reflects a broader constraint in network design, as current strategies mainly rely on homogeneous, bulk-level control of exchange kinetics. In contrast, emerging evidence from dynamic nanocomposites suggests that nanoparticle–polymer interfaces can act as local regulators of *E*_a_, enabling spatially heterogeneous dynamics and partially decoupling exchange behavior from bulk composition.^16–19^

Here, we propose an interfacial kinetic engineering approach in which the surface chemistry of nanoparticles (NPs) is used to modulate *E*_a_ at the nanoscale, providing a new and independent design dimension for tuning *T*_v_. This broader perspective leads to a critical conclusion: *T*_v_ should be understood as a kinetic design parameter rather than an intrinsic material constant. This insight provides an opportunity to move beyond bulk design strategies toward approaches that enable direct and localized control of bond-exchange kinetics, thereby creating new possibilities for application-specific material functionality.

## 2 Definition and Measurement of Topology-Freezing Temperature (*T*_v_)

The understanding of vitrimer dynamics has evolved considerably over the past two decades. While early studies focused primarily on developing new dynamic covalent chemistries, recent work has shifted toward elucidating the molecular mechanisms governing stress relaxation, bond exchange, and network rearrangement. Figure 1 provides a chronological overview of these developments, which are discussed in more detail below.

**Figure 1:**
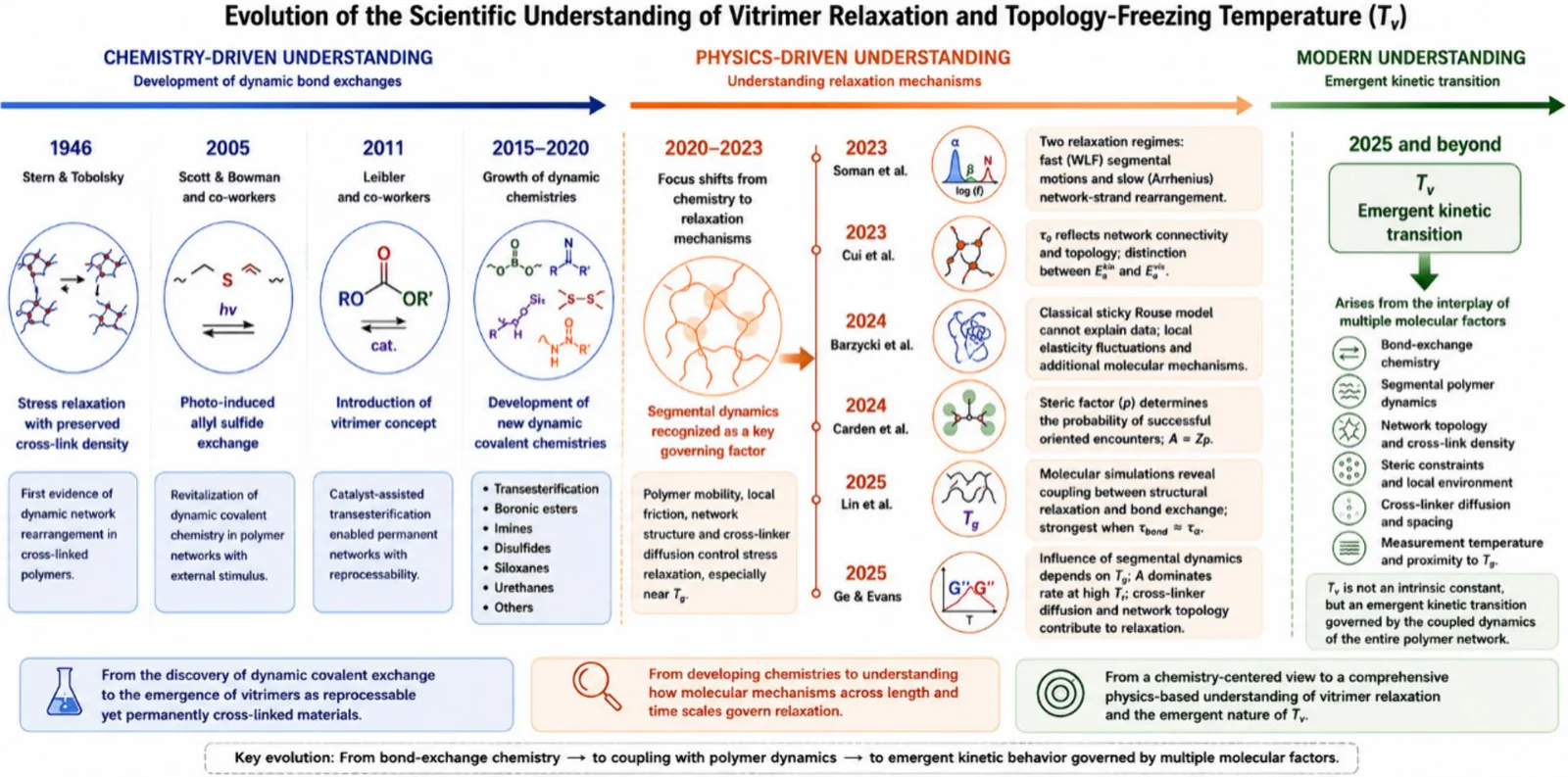
Evolution of the scientific understanding of *T*_v_.

The *T*_v_ has traditionally been interpreted as the characteristic temperature at which dynamic polymer networks transition from a viscoelastic solid to a malleable viscoelastic liquid as a consequence of thermally activated bond-exchange reactions. However, such a definition is fundamentally oversimplified. Unlike the *T*_g_, which is governed by segmental chain mobility and free-volume evolution, *T*_v_ represents a kinetically defined parameter governed by the rate of associative exchange reactions within the network rather than a thermodynamic transition. The *T*_g_ reflects the progressive slowing of segmental chain mobility and the evolution of free volume, while the experimentally determined *T*_g_ depends on the relevant time scale, including the heating or cooling rate, measurement frequency, and observation time. In contrast, *T*_v_ represents a kinetically defined parameter associated with the onset of associative dynamic bond-exchange reactions and the resulting network topology rearrangement. As such, *T*_v_ reflects the interplay between *E*_a_, molecular mobility, and the experimental time scale used for its determination.^4–6^ Importantly, the experimentally determined *T*_v_ strongly depends on the methodology used and should therefore not be regarded as an intrinsic material constant.^20–22^ Because bond-exchange reactions are kinetically controlled, the apparent *T*_v_ is influenced by the selected experimental technique, observation time scale, relaxation criterion, strain or stress conditions, and the thermal history of the sample. Consequently, identical dynamic polymer networks may exhibit substantially different reported *T*_v_ values depending on whether the parameter is derived from viscosity extrapolation, stress relaxation, creep experiments, or dynamic mechanical analysis. Recent studies have increasingly emphasized that *T*_g_ and *T*_v_ originate from fundamentally different physical phenomena and should therefore not be interpreted as directly analogous transition temperatures. This distinction has led to a broader understanding of vitrimer relaxation. Using silyl ether vitrimer networks, Nishimura et al. investigated the relationship between *T*_v_, *T*_g_, and temperature-dependent relaxation behavior. They experimentally demonstrated that vitrimer dynamics cannot be described by a single Arrhenius relationship over the entire temperature range. Instead, relaxation near *T*_g_ is governed predominantly by segmental chain mobility and follows Williams–Landel–Ferry (WLF) behavior, whereas at higher temperatures the dominant mechanism shifts toward Arrhenius-controlled bond-exchange kinetics. Their study further revealed that the relative positions of *T*_v_ and *T*_g_ are polymer-dependent and that *T*_v_ may even occur well below *T*_g_, highlighting that vitrimer dynamics arise from the interplay between polymer mobility and exchange chemistry rather than from a single intrinsic material property. Importantly, apparent *E*_a_ measured in vitrimer networks is significantly higher than that obtained for analogous small-molecule model reactions. The authors attributed this increase to steric hindrance and restricted polymer-chain diffusion within the cross-linked network, indicating that bond-exchange kinetics are governed not only by the intrinsic reaction chemistry but also by the local polymer environment. These findings suggest that factors affecting chain mobility and steric accessibility may significantly influence vitrimer relaxation and the apparent *T*_v_.^23^

Building upon the concept of distinct WLF- and Arrhenius-controlled relaxation regimes, Lee et al. investigated poly­(β-amino ester) covalent adaptable networks to establish a practical rheological criterion for network reprocessing. The authors demonstrated that the temperature dependence of the horizontal rheological shift factor (a_*T*_) is characterized by two distinct regimes: a WLF-controlled regime at lower temperatures, where relaxation is governed primarily by segmental chain mobility, and an Arrhenius-controlled regime at higher temperatures, where dynamic bond-exchange reactions dominate network rearrangement. Based on this transition, the authors introduced an empirical reprocessing temperature (*T*_r_), defined as the crossover between the WLF and Arrhenius fits of the horizontal shift factors. Although *T*_r_ is not proposed as a replacement for *T*_v_, it was described as conceptually similar and represents a practical rheological parameter for identifying the onset of efficient network rearrangement during processing. This study further demonstrated that stress relaxation is governed not only by bond-exchange kinetics but also by the segmental mobility of the polymer network. By comparing different polymer networks under iso-free-volume conditions (*T*_g_ + 100 °C), the authors showed that differences in relaxation behavior become significantly reduced, indicating that the apparent *E*_a_ and relaxation kinetics arise from the combined contributions of segmental dynamics, free volume, exchange chemistry, and the local molecular environment rather than from bond-exchange chemistry alone.^24^

Providing molecular-level support for these experimental observations, Perego and Khabaz employed a hybrid molecular dynamics/Monte Carlo (MD/MC) simulation approach to investigate vitrimer relaxation. Their simulations revealed the existence of two distinct relaxation regimes, with WLF-controlled dynamics dominating at lower temperatures and Arrhenius-controlled bond exchange prevailing at higher temperatures. Importantly, the crossover between these regimes was identified as corresponding to the *T*_v_, providing theoretical evidence that *T*_v_ represents a kinetic transition between diffusion-controlled and reaction-controlled network relaxation rather than a fixed material constant.^25^

Fang et al. further expanded this framework by demonstrating that vitrimer relaxation is additionally governed by network morphology. Using smectic liquid-crystalline elastomer vitrimers, they experimentally confirmed the transition from WLF-controlled relaxation at lower temperatures to Arrhenius-type behavior at higher temperatures, while simultaneously analyzing the rheological response during both heating and cooling. Their results demonstrated that vitrimer dynamics evolve through multiple temperature-dependent regimes and therefore cannot be adequately described by a single relaxation equation. Importantly, the WLF *E*_a_ was shown to reflect polymer backbone stiffness and segmental mobility, whereas the apparent Arrhenius *E*_a_ included not only dynamic bond-exchange kinetics but also the energy required to release transient cross-links from smectic domains, demonstrating that supramolecular organization contributes directly to vitrimer relaxation. Furthermore, stress relaxation above the liquid-crystalline transition was successfully described using a stretched exponential Kohlrausch–Williams–Watts (KWW) function, indicating that relaxation proceeds through a distribution of relaxation times rather than a single characteristic process. The corresponding low stretching exponent (β) revealed pronounced structural heterogeneity, indicating that different regions of the network relax at different rates. These findings suggest that the *E*_a_ governing vitrimer relaxation should be regarded as a local network property, influenced not only by exchange chemistry but also by catalyst distribution, functional-group organization, and the surrounding network morphology.^26^

Hayashi et al. further transformed the interpretation of *T*_v_ by demonstrating that its determination should not rely solely on rheological measurements but instead combine complementary structural and rheological analyses. The authors showed that conventional temperature-ramp creep experiments may yield apparent *T*_v_ values that depend on the applied stress, highlighting the influence of measurement conditions and nonlinear deformation. To overcome this limitation, they combined stress-free temperature-dependent small-angle X-ray scattering (SAXS) with frequency-sweep rheology. Structurally, *T*_v_ was identified as the temperature at which an anomalous increase in the interdomain spacing occurred, whereas rheologically it corresponded to the crossover from WLF-controlled to Arrhenius-controlled horizontal shift factors. Remarkably, both approaches yielded nearly identical *T*_v_ values, providing strong evidence that the *T*_v_ has a structural origin and represents a transition in the dominant relaxation mechanism rather than merely a viscosity-derived parameter. This work establishes a new framework for interpreting *T*_v_, where structural evolution and network dynamics are considered together to provide a more physically meaningful description of vitrimer relaxation. Collectively, these findings support the emerging view that *T*_v_ should not be interpreted as a unique intrinsic material constant, but rather as a kinetic transition reflecting the crossover from segmental mobility-controlled to bond-exchange-controlled network relaxation, governed by the interplay between polymer dynamics, network morphology, and local exchange kinetics.^20,21,27^

In dynamic polymers, stress relaxation and network rearrangement often follow Arrhenius-type behavior, where the characteristic relaxation time (τ) decreases exponentially with increasing temperature.^6,20,28^ This kinetic nature is typically expressed as

$$\tau =\tau_{0}\;\exp\left(\frac{E_{a}}{RT}\right)$$

where τ is the relaxation time, τ_0_ is the pre-exponential factor, *E*_a_ is the activation energy, *R* is the gas constant, and *T* is the absolute temperature.

Consistent with this macromolecular interpretation, accumulating experimental evidence indicates that stress relaxation cannot always be explained solely by the intrinsic kinetics of bond-exchange reactions. In particular, mechanistic studies of imine-based dynamic covalent networks demonstrated that efficient network rearrangement depends on both the exchange mechanism and the segmental mobility of the surrounding polymer matrix. These findings revealed that polymer relaxation and bond-exchange kinetics are intrinsically coupled, leading Carden et al. to propose an extended description of vitrimer relaxation in which the characteristic τ depends on both segmental polymer dynamics and bond-exchange kinetics:^29^

$$\tau_{r}\left(T\right)={\sigma \tau }_{\alpha }\left(T\right)\;\exp\left(\frac{E_{a}}{RT}\right)$$

where τ_r_ is the macroscopic stress-relaxation time, τ_α_ is the segmental (α) relaxation time of the polymer matrix, σ is the coupling exponent describing the degree of interaction between segmental mobility and bond exchange, and *E*_a_ is the apparent activation energy associated with the exchange reaction. In this framework, the first term represents the contribution of polymer segmental dynamics, whereas the Arrhenius term describes the intrinsic kinetics of associative bond-exchange reactions.

While the extended Arrhenius framework incorporates the coupling between segmental mobility and bond-exchange kinetics, it does not by itself provide a molecular-level description of vitrimer flow. From a macromolecular perspective, vitrimer rheology arises from the coupling between polymer chain relaxation, network-strand dynamics, and dynamic cross-link exchange. A foundational framework for describing this coupling is the Sticky Rouse model, in which dynamic cross-links are treated as transient “sticky” constraints superimposed on the Rouse relaxation of polymer strands. Ricarte and Shanbhag further advanced this concept through the Inhomogeneous Rouse Model, which accounts for heterogeneous strand environments and relaxation pathways in dynamic networks.^30^ Together with recent structure–dynamics–rheology descriptions of vitrimers,^31^ this study shows that short-time relaxation is governed primarily by backbone or segmental dynamics, whereas long-time relaxation reflects the coupled effects of network-strand relaxation and cross-link exchange. Consequently, the apparent relaxation time, rheological activation energy, and *T*_v_ should be interpreted as emergent quantities arising from exchange chemistry, macromolecular mobility, and network architecture rather than as direct measures of bond-exchange kinetics alone.

Lin et al. further expanded the current understanding of vitrimer dynamics by combining molecular dynamics simulations with theoretical analysis to investigate the coupling between structural relaxation, bond-exchange dynamics, and *T*_g_. Their results demonstrated that bond exchange cannot be considered independently of polymer structural relaxation, as both processes become increasingly coupled near the *T*_g_. Consequently, the apparent *E*_a_ and characteristic τ emerge from the combined effects of bond-exchange kinetics, segmental dynamics, and network relaxation rather than from the intrinsic reaction barrier alone. These findings further support the view that vitrimer relaxation arises from the interplay between molecular-exchange reactions and polymer dynamics, reinforcing the interpretation of *T*_v_ as an emergent kinetic transition rather than an intrinsic material constant.^32^

The interpretation of the Arrhenius equation was further refined by Carden et al., who demonstrated that bond-exchange kinetics are governed not only by the *E*_a_ but also by the steric factor (*p*), which describes the probability that reactive functional groups adopt the correct orientation for successful bond exchange. Accordingly, the pre-exponential factor (*A*) should not be regarded solely as the collision frequency but instead expressed as^33^

A∝Zp

where *Z* is the collision frequency and *p* is the steric factor. Importantly, *p* should be distinguished from conventional steric effects that contribute to the activation-energy barrier: whereas steric hindrance may contribute to the intrinsic activation barrier, the steric factor (*p*) describes the probability that reactive groups adopt the correct orientation for bond exchange. Using model dynamic covalent networks containing boric ester functionalities, together with literature data for boric-, boronic-, and imine-based polymers, the authors showed that relatively low activation-energy barriers may still result in slow bond rearrangement when the steric factor is exceptionally small. For boric- and boronic-ester exchange, estimated *p* values ranged from approximately 10^–11^ to 10^–8^. These findings provide a physical explanation for the unusually large time prefactors observed in dynamic polymer networks and show that vitrimer relaxation depends not only on the activation-energy barrier but also on steric accessibility, collision frequency, segmental mobility, and network architecture.^33^

Further insight into the physical interpretation of the Arrhenius parameters was provided by Soman et al.,^35^, who investigated boronic ester-based ethylene vitrimers using broadband dielectric spectroscopy (BDS) in combination with rheology. By identifying three distinct relaxation processes—α-relaxation associated with segmental chain mobility, β-relaxation related to localized molecular motions, and a normal mode corresponding to network-strand dynamics—the authors demonstrated that the characteristic τ is governed not only by bond-exchange kinetics but also by polymer segmental dynamics. Notably, the normal mode closely coincided with the rheological crossover time, indicating that macroscopic flow is controlled by network-strand exchange at elevated temperatures. A key finding of this work is that vitrimer relaxation proceeds through two distinct dynamic regimes. At sufficiently high temperatures, τ follows Arrhenius behavior dominated by bond-exchange reactions.

However, as the temperature approaches *T*_g_, increasing segmental friction and slowing polymer-chain dynamics lead to positive deviations from Arrhenius behavior. This indicates that τ is an emergent property arising from the combined influence of bond-exchange reactions, segmental mobility, and network relaxation rather than a direct measure of exchange kinetics alone. Importantly, although bond-exchange-mediated relaxation is affected by the surrounding polymer dynamics, its temperature dependence remains substantially weaker than that of the more temperature-sensitive α-relaxation, indicating partial dynamic decoupling between the two processes. Consequently, the Arrhenius parameters, including the apparent *E*_a_ and the pre-exponential factor (τ_0_), should be interpreted in the context of both bond-exchange kinetics and polymer segmental dynamics. Together with the extended kinetic model proposed by Carden et al.,^29,33^ these findings mark an important shift from the classical interpretation of vitrimer relaxation, where τ was considered to reflect only bond-exchange kinetics, toward a framework in which macroscopic relaxation emerges from the combined contributions of exchange chemistry, segmental dynamics, and network relaxation. From this perspective, the apparent *T*_v_ should be interpreted as an operational consequence of these coupled molecular processes rather than of exchange chemistry alone. It represents a practical threshold at which bond exchange becomes sufficiently fast to allow measurable relaxation within a given observation window. Accordingly, *T*_v_ is inherently method- and time scale-dependent, and may vary significantly even for chemically similar polymers when measured under different experimental conditions.^4,20,28,34,35^

The physical interpretation of the Arrhenius parameters was further examined by Cui et al., who employed hybrid Monte Carlo/Molecular Dynamics (MC/MD) simulations to directly compare associative (vitrimer) and dissociative covalent adaptable networks while maintaining an identical network architecture. The simulations showed that, although both types of dynamic polymer networks follow Arrhenius behavior, the physical meaning of the Arrhenius parameters differs fundamentally. In associative networks, the pre-exponential factor (τ_s_^0^) remains nearly constant because the cross-link density is preserved during bond exchange, whereas in dissociative networks τ_s_^0^ varies owing to the progressive loss of network connectivity. These findings demonstrate that τ_0_ is not merely a mathematical fitting parameter but also reflects the structural state of the polymer network. Furthermore, the authors distinguished between the intrinsic kinetic activation energy (*E*_a_^kin^) and the apparent activation energy governing viscous flow (*E*_a_^vis^). In vitrimers, *E*_a_^vis^ depends not only on bond-exchange kinetics but also on network topology and the spatial constraints associated with four-body-exchange events. Consequently, the apparent *E*_a_ reflects the combined influence of exchange chemistry and network architecture, highlighting that vitrimer rheology is governed by network reorganization rather than bond chemistry alone.^36^

Barzycki et al. further advanced the understanding of vitrimer relaxation by experimentally identifying two distinct dynamic regimes operating over different time scales. The fast, high-frequency regime was attributed to segmental motions of the polymer backbone and exhibited Williams–Landel–Ferry (WLF) temperature dependence. By contrast, the slow, low-frequency regime was associated with terminal network relaxation and exhibited Arrhenius-type behavior with an *E*_a_ that was independent of temperature and cross-linker concentration. The authors termed this long-time response the slow Arrhenius process (SAP). Importantly, the SAP exhibited a substantially weaker temperature dependence than predicted by the sticky Rouse model. Whereas the model assumes that long-time relaxation combines the temperature dependences of segmental motion and cross-link exchange, the experimental results indicated that the cross-link-exchange attempt time was not directly coupled to segmental relaxation. The molecular origin of the SAP nevertheless remains unresolved. Cross-linker diffusion, polymer-matrix effects, and local elasticity fluctuations were proposed as three possible mechanisms that may partially explain the observed behavior. Accordingly, the rheological *E*_a_ of the slow process should be regarded as an effective parameter associated with long-time network relaxation rather than as a direct measurement of the intrinsic bond-exchange barrier.^37^

Lin et al. further expanded the molecular-level understanding of vitrimer dynamics by combining coarse-grained molecular dynamics simulations with statistical mechanical theory to investigate the coupling among segmental structural relaxation, bond-exchange dynamics, and *T*_g_. Their results showed that bond exchange and segmental relaxation become coupled upon cooling, particularly when the bond-exchange time approaches the Kuhn-segment α-relaxation time. This coupling is reciprocal: slow segmental dynamics can limit bond exchange, whereas rapid bond exchange can accelerate segmental relaxation by releasing cross-link constraints, especially at higher cross-link densities. Consequently, the observed relaxation times reflect the combined effects of exchange kinetics, segmental mobility, cross-link density, and network constraints rather than bond-exchange chemistry alone.^38^ Although *T*_v_ was not directly evaluated in this study, these results are consistent with its broader interpretation as an emergent, time scale-dependent kinetic parameter rather than an intrinsic material constant.

Ge and Evans further demonstrated that vitrimer relaxation is strongly influenced by the interplay between bond-exchange kinetics and polymer segmental dynamics. By systematically varying the polymer backbone and cross-linker chemistry in imine vitrimers, they showed that the relative contribution of these processes depends on the *T*_g_ of the network. At temperatures well above *T*_g_, terminal relaxation is influenced predominantly by local bond-exchange kinetics, whereas closer to *T*_g_, slowing segmental dynamics and cross-linker diffusion increasingly limit network rearrangement. Cross-linker flexibility and polarity also affected both *T*_g_ and the terminal relaxation rate. Consequently, the measured rheological parameters such as τ and the apparent *E*_a_, should be interpreted as emergent quantities arising from the combined effects of exchange chemistry, polymer dynamics, cross-linker mobility, and network architecture rather than as intrinsic material constants. Although *T*_v_ was not directly determined, these findings are consistent with its interpretation as an emergent kinetic parameter governed by the same coupled processes.^39^

Recent advances have fundamentally reshaped the classical understanding of vitrimer dynamics. Rather than being governed by a single activation energy or bond-exchange reaction, network relaxation emerges from the cooperative interplay of multiple molecular processes, including dynamic bond exchange, polymer segmental mobility, steric constraints, cross-linker diffusion, and network topology. The relative importance of these mechanisms evolves with temperature, giving rise to distinct relaxation regimes. Consequently, kinetic descriptors such as τ, apparent *E*_a_, pre-exponential factor (*A* or τ_0_), and the *T*_v_ should be regarded as effective, network-level quantities rather than intrinsic material constants. This perspective provides a more comprehensive framework for interpreting vitrimer rheology and for designing dynamic polymer networks with tailored processing and service performance. Within a temperature range where relaxation follows approximately Arrhenius behavior, the apparent *T*_v_ is strongly influenced by the apparent *E*_a_ and the corresponding pre-exponential factor. Because the τ depends exponentially on temperature, changes in these kinetic parameters can substantially shift the temperature at which network rearrangement becomes observable within a specified experimental time scale. Thus, *T*_v_ is not an independent transition temperature, but an operationally derived quantity defined by the relaxation criterion and observation window. Factors that alter exchange chemistry, catalytic activity, segmental mobility, steric accessibility, or cross-linker diffusion may therefore affect *T*_v_ by modifying the apparent *E*_a_, the kinetic prefactor, or both. At otherwise comparable kinetic conditions, a lower apparent *E*_a_ generally promotes faster relaxation and may reduce the apparent *T*_v_, whereas a higher *E*_a_ shifts measurable network rearrangement toward higher temperatures.^4,20,28,34^ Several experimental approaches are used to estimate *T*_v_, each associated with inherent limitations (Table 1), including viscosity extrapolation, stress relaxation, creep, and thermal analysis. However, these methods yield different values due to their dependence on measurement conditions and analysis criteria. Standardized reporting of *E*_a_, relaxation times, and experimental protocols is therefore essential for meaningful comparison across vitrimers.^20,21^

**Table 1:** Methods for Estimating the *T*_v_ ^a^

Method	Principle	Output	Strength	Key Limitation	Ref
Viscosity extrapolation (η = 10^12^ Pa·s)	Extrapolation of η(*T*) to η = 10^12^ Pa·s	Apparent *T*_v_	Simple conceptual analogy to *T*_g_	Requires large extrapolation; unreliable near *T*_g_ and in semicrystalline polymers (e.g., PCL)	^5^,^28^
Stress relaxation (rheology/DMA)	τ_(*T*)_ measured; *T*_v_ defined at characteristic τ (10^2^–10^3^ s)	τ(*T*), *E*_a_, apparent *T*_v_	Direct link to network dynamics; enables Arrhenius analysis	Arbitrary τ criterion; sensitive to strain, frequency, and thermal history	^20^
Creep experiments	Time-dependent deformation under constant stress	Apparent transition temperature/creep onset	Simple experimental setup	Strong stress dependence; poor reproducibility	^47^
Dilatometry/thermal expansion/TMA	Change in thermal expansion or dimensional response	Apparent transition point	Complementary method	Low sensitivity; morphology-dependent	^46^
Fluorescence-based detection (AIE luminogens)	Probe response to local mobility/topology change	Apparent/probe-detected *T*_v_	Highly sensitive probe capable of detecting local network mobility and nanoscale topology transitions without applying macroscopic deformation	Specialized method; limited general adoption and comparability across vitrimer	^22^

a Stress relaxation, small-amplitude oscillatory shear (SAOS), creep, and frequency-sweep measurements constitute complementary rheological techniques used to determine relaxation times or shift factors. These experimental data are subsequently analyzed using Arrhenius relationships to extract *E*_a_ and estimate the *T*_v_. Thus, Arrhenius analysis should be regarded as part of the rheological analysis workflow rather than as a standalone experimental method. Complementary techniques, including dielectric spectroscopy (DS), small-angle X-ray scattering (SAXS), dynamic mechanical analysis (DMA), differential scanning calorimetry (DSC), X-ray diffraction (XRD), atomic force microscopy (AFM), and spectroscopic ellipsometry, do not determine T_v_ directly but provide valuable structural and molecular insight into the relaxation processes governing vitrimer dynamics.

One of the most widely used definitions is based on viscosity extrapolation, where *T*_v_ is defined as the temperature at which the viscosity reaches 10^12^ Pa·s, by analogy with the conventional viscosity criterion used for *T*_g_. While conceptually simple, this approach requires extrapolation over multiple orders of magnitude and assumes ideal Arrhenius behavior, which may become problematic near *T*_g_ or in semicrystalline polymers such as PCL-based dynamic polymers.^4,6^ Consequently, viscosity-derived *T*_v_ values may be highly sensitive to the fitting range, rheological model, and selected kinetic criterion.

In semicrystalline PCL-based dynamic polymers, crystalline domains act as reversible physical cross-links below the melting temperature of PCL and can suppress chain relaxation until these domains soften or melt. As a result, viscosity- or stress-relaxation-derived *T*_v_ values may reflect the combined effects of crystal softening/melting and dynamic covalent exchange rather than bond-exchange kinetics alone. Arrhenius extrapolation in such dynamic polymers should therefore be interpreted with caution and complemented by thermal, structural, or rheological characterization to distinguish crystallinity-controlled softening from chemically induced network rearrangement.^40–42^

Rheological determination of *T*_v_ commonly involves complementary techniques such as stress relaxation, small-amplitude oscillatory shear, creep, and frequency-sweep measurements combined with time–temperature superposition. These methods provide access to different characteristic times and kinetic descriptors, including stress-relaxation times, rheological crossover times, shift factors, terminal viscosity, and apparent activation energies. However, the resulting τ depends on its operational definition, such as a selected stress-decay criterion, the *G*′ = *G*″ crossover, or a terminal-flow time scale. Stress relaxation, creep, oscillatory rheology, and viscosity measurements should therefore be regarded as complementary rather than directly interchangeable descriptions of vitrimer dynamics. Recent studies further indicate that the characteristic relaxation time, apparent *E*_a_, kinetic prefactor, and apparent *T*_v_ are effective network-level quantities arising from the coupled contributions of bond-exchange kinetics, segmental dynamics, steric constraints, cross-linker diffusion, network topology, and local structural organization.^43–45^ Structural methods may therefore complement rheological measurements, particularly in phase-separated or morphologically complex networks where structural reorganization accompanies changes in relaxation behavior.^4,46–48^

A recent advance in the characterization of *T*_v_ is the use of spectroscopic ellipsometry, which enables the stress-free determination of topology transitions by monitoring temperature-dependent thickness changes in vitrimer thin films. Unlike conventional rheological methods, which probe the macroscopic mechanical response of bulk samples, ellipsometry directly detects changes in local relaxation dynamics without applying an external mechanical deformation. Consequently, this approach is particularly promising for investigating nanoscale interfacial regions and supported vitrimer films, where localized network dynamics may not be adequately resolved by bulk rheological measurements. Such local characterization methods are expected to become increasingly important for validating interfacial kinetic engineering strategies in vitrimer nanocomposites.^49^

Reliable interpretation of *T*_v_ consequently requires detailed reporting of the measurement technique, deformation conditions, temperature range, observation time scale, relaxation criterion, fitting procedure, and associated kinetic parameters.

The variability and methodological dependence of the approaches summarized in Table 1 highlight that *T*_v_ is not a uniquely defined intrinsic material parameter, but an operational quantity that depends on the selected experimental method, analysis criterion, and observation time scale. This methodological variability complicates both the comparison of reported *T*_v_ values across different vitrimers and the systematic tuning of dynamic behavior using conventional bulk design strategies.

## 3 Activation Energy as a Design Parameter in PCL-Based Dynamic Polymers

*E*_a_ represents a key parameter controlling the kinetics of bond-exchange reactions in dynamic polymers and largely defines the temperature range over which network rearrangement becomes accessible. In PCL-based dynamic polymers, *E*_a_ is not an intrinsic property of the polymer structure, but rather an emergent parameter governed by chemical environment, catalyst activity, and network structure.^20,21,28^

### 3.1 Catalytic Control of Exchange Kinetics in PCL-Based Dynamic Polymers

The bond-exchange kinetics of PCL-based dynamic polymers are strongly influenced by catalytic effects, which influence relaxation by modifying the apparent *E*_a_, kinetic prefactor, or both. In transesterification-based networks, catalysts play a central role by lowering the energy barrier for ester-exchange reactions, thereby accelerating stress relaxation and enabling dynamic network rearrangement without altering the permanent cross-linked structure.^4,50,51^ Catalyst identity strongly influences *E*_a_, and different catalysts exhibit markedly different efficiencies in promoting transesterification, leading to substantial variation in relaxation kinetics (Table 2). Nucleophilic catalysts such as triphenylphosphine (PPh_3_) can result in relatively low *E*_a_ (≈40–50 kJ/mol), enabling rapid stress relaxation and lower effective *T*_v_. In contrast, Lewis acid catalysts such as zinc acetate typically exhibit intermediate *E*_a_ (≈80–90 kJ/mol), while strong organic bases such as 1,5,7-triazabicyclo[4.4.0]­dec-5-ene (TBD) can lead to higher *E*_a_ (≈100–110 kJ/mol), depending on the reaction pathway and stabilization of intermediates. Metal-based catalysts, including tin­(II) 2-ethylhexanoate (Sn­(Oct)_2_) and dibutyltin dilaurate (DBTDL), are widely used due to their balance of catalytic activity, stability, and compatibility with polyesters, typically yielding *E*_a_ values in the range ≈60–90 kJ/mol.^50–53^ Catalyst concentration also modulates exchange kinetics. At low catalyst concentrations, limited availability in the network results in slow exchange kinetics and poor reprocessability. Conversely, higher catalyst loadings generally accelerate stress relaxation and may lower the apparent *T*_v_, although this does not necessarily result from a decrease in *E*_a_. Importantly, this effect is achieved without significantly altering the *T*_g_, demonstrating that bond-exchange dynamics can be decoupled from conventional thermomechanical properties.^11,50^

**Table 2:** Influence of Catalyst Type on *E*_a_, *T*_v_, and Relaxation Kinetics in Transesterification-Based Dynamic Polymers

Catalyst	Catalyst Type	Typical *E*_a_ (kJ/mol)	Effect on Relaxation Rate (τ)	Effect on *T*_v_	Mechanistic Role	Key Insight	Ref
Triphenylphosphine (PPh_3_)	Nucleophilic/organophosphine	∼40–50	Very fast relaxation (low τ)	Strong ↓ *T*_v_	Activates ester exchange via nucleophilic catalysis	Enables low activation energy and fast network rearrangement	^28^
Zinc acetate (Zn(OAc)_2_)	Lewis acid	∼80–90	Moderate relaxation	Moderate ↓ *T*_v_	Coordinates ester groups, facilitating exchange	Balanced catalytic activity and stability	^6^
Triazabicyclodecene (TBD)	Strong organic base	∼100–110	Slower relaxation (higher τ than PPh_3_)	Higher *T*_v_	Strong base enhances nucleophilicity but also stabilizes intermediates	High activity but can lead to higher apparent *E*_a_	^4^
Tin(II) 2-ethylhexanoate (Sn(Oct)_2_)	Metal catalyst	∼70–90	Moderate to fast relaxation	Moderate ↓ *T*_v_	Promotes transesterification via coordination and activation of ester bonds	Widely used due to stability and efficiency	^34^
Dibutyltin dilaurate (DBTDL)	Organotin catalyst	∼60–80	Fast relaxation	Lower *T*_v_	Highly active transesterification catalyst	Efficient but potential toxicity issues	^6^
No catalyst		≫120	Extremely slow/negligible relaxation	Very high *T*_v_	No activation of exchange reactions	System behaves as permanent thermoset	^28^

Despite this tunability, catalytic control remains inherently a bulk level strategy. Changes in catalyst type or concentration uniformly affect the entire network, resulting in spatially homogeneous exchange kinetics.^6^ Reported *E*_a_ values are dynamic-dependent *E*_a_ values and may vary with polymer matrix, network architecture, catalyst concentration, and measurement protocol.

These results clearly demonstrate that catalyst chemistry strongly influences *E*_a_ and relaxation kinetics in dynamic polymers, thereby defining *T_v_* as a kinetically tunable parameter controlled at the bulk level.

For biomedical applications, catalyst selection must consider not only exchange efficiency but also cytocompatibility, migration, and leaching. Although tin-based catalysts such as Sn­(Oct)_2_ and DBTDL are highly effective for transesterification, residual organotin or tin-containing species may raise cytotoxicity concerns, particularly in degradable or implantable materials.^54^ Therefore, biomedical PCL-based dynamic polymers should prioritize catalyst systems with improved biological profiles, including catalyst-free exchange chemistries, internal or neighboring-group catalysis, immobilized catalytic groups, and potentially more biofriendly Brønsted acid, phosphate-based, or biologically relevant metal-based catalysts.^55–57^ Immobilizing catalytic groups at NPs surfaces may further reduce catalyst migration while enabling localized exchange kinetics.

However, any proposed catalyst must be evaluated through extraction and leaching studies, in vitro cytocompatibility testing according to ISO 10993–5, and degradation-product characterization before biomedical relevance can be claimed.^54,58^

### 3.2 Role of Dynamic Covalent Chemistry

Beyond catalysis, the nature of the dynamic bond plays a major role in determining *E*_a_.^4,59^ Imine-based networks typically show relatively low *E*_a_ (∼23–81 kJ/mol), while boronic ester networks exhibit intermediate values (∼44–61 kJ/mol), although lower values have also been reported in specific dynamic polymer (∼18.2 kJ/mol). In contrast, transesterification-based dynamic PCL networks generally range between ∼45 and 90 kJ/mol, reflecting the higher energy barrier associated with transesterification between ester and hydroxyl groups.^4,6,60–65^ These differences indicate that *E*_a_ is strongly influenced by the exchange mechanism, although catalyst effects, network architecture, and molecular mobility also contribute. As a result, the selection of dynamic chemistry provides a fundamental design tool for tuning bond-exchange kinetics,^66^.^67^ Taken together, these observations indicate that, although PCL-based dynamic polymers exhibit well-defined dynamic behavior and tunable kinetics, precise and independent control over the temperature and spatial location of exchange processes remains challenging.^1–3,13,14^ This reinforces the need for alternative design strategies that enable more refined and potentially localized control of *E*_a_ and *T*_v_.^16–18^

### 3.3 Influence of Network Architecture

Network architecture further modulates exchange kinetics by influencing chain mobility and accessibility of reactive groups,^67,68^ Increased hydroxyl group concentration promotes transesterification by increasing the availability of nucleophilic sites and probability of exchange reactions.^51^ The influence of cross-link density is more complex: higher cross-link densities may restrict segmental mobility and slow network relaxation but may also increase the local concentration of exchangeable functionalities. The resulting effect is therefore system-dependent.^69^ Network architecture may influence the apparent *E*_a_, kinetic prefactor, and characteristic relaxation time by modifying the local molecular environment and configurational constraints associated with exchange.^67,68^ It should therefore be regarded as a network-level regulator of dynamic behavior rather than merely an indirect modifier of bond-exchange kinetics.

### 3.4 Internal Catalysis and Neighboring Group Effects

Beyond externally added catalysts, bond-exchange kinetics in dynamic polymer networks can also be regulated by internal catalytic effects arising from the network structure itself. Neighboring group participation can enhance exchange kinetics through the local activation of reactive sites. In polyester-based polymers, hydroxyl-rich environments are known to promote transesterification by increasing the availability of nucleophilic species, while activated ester functionalities can enhance the electrophilicity of the carbonyl group, facilitating the bond exchange. Such effects have been shown to enable efficient exchange reactions even in the absence of externally added catalysts, demonstrating that catalytic activity can be encoded directly into the network architecture.^70,71^ These internal catalysts provide molecularly integrated catalytic activity and can reduce issues related to catalyst migration, deactivation, or leaching while enabling more controlled and predictable exchange kinetics. Recent studies have demonstrated that such approaches can significantly enhance relaxation behavior and enable vitrimer-like dynamics without the need for free catalysts. These findings highlight that *E*_a_ can be modulated not only through external catalyst addition but also through molecular design of the network itself. Importantly, both neighboring group effects and internal catalysis provide molecular-scale regulation of the exchange kinetics. However, this control remains inherently coupled to the polymer structure and lacks the flexibility required for independent and spatially resolved tuning of dynamic behavior.^70^

### 3.5 Limitations of Bulk Kinetic Control

Taken together, these observations demonstrate that *E*_a_ and, consequently, *T*_v_ are largely influenced by bulk chemical parameters, including exchange chemistry, catalyst type, and network architecture.^51,72,73^ While these strategies provide effective means to tune bond-exchange behavior, they inherently assume spatially uniform kinetics throughout the material.^72,73^

As a result, any modification of *E*_a_ affects the entire network simultaneously, limiting the ability to achieve localized, gradient, or application-specific control over the dynamic behavior. This limitation becomes particularly important in advanced applications such as self-healing biomaterials, adaptive implants, and stimuli-responsive materials, where selective activation of exchange reactions would be highly desirable.^72,74,75^

These constraints highlight the need for alternative design strategies capable of introducing localized control over bond-exchange kinetics beyond conventional bulk approaches.^16–18^

## 4 State of the Art: PCL-Based Polymer Networks and Their Nanocomposites

The development of PCL-based dynamic polymers has primarily relied on the introduction of associative dynamic covalent chemistries into the polyester networks. Among these, transesterification is the most extensively investigated mechanism, in which exchange reactions occur between ester and hydroxyl groups within the network, typically in the presence of catalysts such as Sn­(Oct)_2_, DBTDL, zinc salts, or organic bases such as TBD. These catalysts play a central role in modifying the apparent *E*_a_ and enabling dynamic bond exchange without disrupting network connectivity.^4,6,52^

Selected transesterification-based PCL dynamic networks have exhibited *E*_a_ in the range of approximately 70–90 kJ/mol, resulting in stress relaxation and network rearrangement at elevated temperatures, often in the range 80–120 °C. While reprocessability and self-healing are well established, *T*_v_ is rarely explicitly reported and is typically inferred indirectly. Consequently, precise control over *T*_v_ remains limited and is governed primarily by global network parameters such as catalyst type, concentration, and cross-link density.^1–4,34^

Alternative dynamic chemistries have been explored to reduce activation barriers and enable more efficient exchange kinetics. Selected boronic ester-based PCL networks exhibit apparent *E*_a_ in the range of 44–62 kJ/mol and can undergo bond exchange without the need for external catalysts due to intrinsic bond reactivity.^14^ An imine-based dynamic PCL material demonstrates even lower *E*_a_ (≈26–48 kJ/mol), while disulfide exchange enables dynamic rearrangement at comparatively mild temperatures.^12^.^13^ Collectively, these studies demonstrate the nature of the exchange chemistry is a major determinant of the apparent *E*_a_ and macroscopic dynamic response. However, the influence of the PCL backbone, including its segmental mobility and semicrystalline morphology, should not be neglected.^67^

Despite this chemical diversity, currently reported dynamic PCL-based polymers remain fundamentally governed by bulk-level design strategies resulting in spatially homogeneous exchange kinetics and limited ability to achieve localized or programmable control.^4,17,51^

### 4.1 Dynamic Polymer Nanocomposites: General Effects of Nanoparticles

Combining dynamic covalent bonds with NPs reinforcement represents an emerging strategy for tailoring mechanical performance, relaxation kinetics, and reprocessability. While this concept has been extensively explored in various dynamic thermosets, its translation to PCL-based dynamic composites remains highly limited. An increasing number of studies demonstrate that NPs play a multifaceted role in dynamic polymer composites, extending far beyond conventional mechanical reinforcement. In dynamic nanocomposites, NPs influence not only macroscopic properties such as stiffness and strength but also the kinetics of dynamic bond exchange, stress relaxation behavior, recyclability, and apparent *T*_v_.^16^

A wide range of NPs has been explored, including inorganic oxides, such as silica, carbon-based materials such as graphene, graphene oxide, and carbon nanotubes, bioderived nanostructures such as cellulose nanocrystals, and nanofibrils, and layered nanoclays.^71,76–85^ These fillers have been incorporated over a broad concentration range, from low loadings of approximately 0.1–1.0 wt % for high-aspect-ratio carbon nanomaterials to significantly higher contents, in some cases up to ∼40 wt %, for inorganic fillers such as silica, depending on the targeted balance between reinforcement and processability.^16,18,86^

A defining parameter governing dynamic nanocomposite performance is NP surface functionalization, which controls the interfacial compatibility, dispersion, and interactions with the dynamic network. Functionalized silica can either accelerate or retard relaxation depending on surface groups, while hydroxyl-rich fillers such as cellulose nanocrystals can participate in transesterification reactions by providing additional reactive sites, thereby increasing the density of exchangeable sites and accelerating bond-exchange kinetics.^71,76,87,88^ Similarly, graphene-based fillers modified with reactive functionalities enhance interfacial adhesion, increase *T*_g_, and improve self-healing efficiency.^84,89^

Importantly, NPs may also influence apparent relaxation kinetics and topology-freezing behavior of dynamic networks. Catalytically active or polar interfaces tend to accelerate exchange reactions and reduce effective *E*_a_, whereas rigid or strongly interacting interfaces restrict chain mobility, slow relaxation, and shift apparent *T*_v_ to higher temperatures.^18,88^ In addition, certain functional NPs enable localized activation mechanisms (e.g., photothermal or magnetic heating), allowing spatially selective acceleration of bond exchange.^90,91^

Beyond kinetic effects, dynamic nanocomposites enable advanced recycling strategies. The combination of dynamic covalent networks and NP facilitates mechanochemical upcycling, repeated reprocessing, and in some cases recovery of NP through mild dissolution processes.^16,18,92^ However, these advantages are accompanied by challenges, including restricted chain mobility due to strong interfacial interactions, increased processing temperatures associated with higher *T*_v_, and difficulties in achieving uniform dispersion at high filler loadings.^88,93^

Collectively, these findings establish that NPs in dynamic polymers are not merely reinforcing agents but active modulators of network dynamics. Through control of surface chemistry, dispersion, and loading, NP–dynamic polymer interfaces define the local chemical and physical environment in which dynamic bond exchange occurs. NPs therefore provide a multidimensional strategy for tuning the apparent *E*_a_, stress-relaxation behavior, and apparent *T*_v_, while simultaneously modifying mechanical performance and reprocessability.^16^

Table 3 highlights that NP effects are highly dependent on the dynamic chemistry and governed by the interplay between interfacial chemistry, dispersion, and network mobility, emphasizing the concept that NP–dynamic polymer interfaces act as local regulators of bond-exchange dynamics.

**Table 3:** Representative Reported Effects of Nanofiller Type, Surface Chemistry, and Loading on Vitrimer Properties and Dynamics

Nanofiller type	Typical loading	Surface functionalization	Effect on mechanical/thermal properties	Effect on apparent *E*_a_/relaxation	Effect on apparent *T*_v_	Mechanistic interpretation	Key references
Silica (SiO_2_, nanosilica)	up to ∼40 wt %	Epoxy- or thiol-functionalized (EP, SH silica)	↑ Modulus, strength, thermal stability	SH: faster relaxation; EP: slower relaxation	↓ or ↑ (depending on surface)	Covalent bonding + interfacial polarity control mobility and exchange accessibility	^76,87,88^
Graphene/GO/rGO	∼0.1–10 wt %	Silane, disulfide, epoxy-functionalized	↑ Strength, *T*_g_, conductivity, thermal stability	Can accelerate or restrict relaxation	↓ (catalytic/polar) or ↑ (confinement)	High surface area; interfacial confinement vs catalytic/π interactions	^77,84,89,94^
Carbon nanotubes (CNTs)	∼0.1–5 wt %	Often functionalized (oxidized, grafted)	↑ Mechanical reinforcement, conductivity	Slight acceleration or restriction depending on dispersion	↔/↑	Percolation network; mobility restriction dominates at higher loading	^77,81,95^
Cellulose nanocrystals (CNC, CNF)	∼1–20 wt %	Native −OH or grafted (e.g., PCL shell)	↑ Modulus, toughness; improved compatibility	↓ *E*_a_ (accelerated exchange)	↓ *T*_v_	Hydroxyl-rich surfaces provide additional exchange sites (transesterification)	^71,96–99^
Nanoclays (montmorillonite, halloysite)	∼1–10 wt %	Organo-modified or pristine	↑ Barrier properties, stiffness	Slower relaxation	↑ *T*_v_	Layered structure induces confinement and restricts chain mobility	^18^
Magnetic nanoparticles (Fe_3_O_4_, etc.)	∼1–10 wt %	Often surface-coated (silica, polymers)	↑ Mechanical + stimuli-responsive behavior	Accelerated relaxation under an alternating magnetic field	↓ (locally under stimulus)	Local heating (magnetic/inductive) enables spatial activation of exchange	^91,100^
Functionalized nanoparticles (general)	variable	Catalytic groups (amines, acids, metal sites)	Reinforcement + multifunctionality	↓↓ *E*_a_ (strong acceleration)	↓↓ *T*_v_	Local catalytic environment lowers transition state barrier	^16^

### 4.2 Interfacial Polymer Physics Governing Bond-Exchange Kinetics

To understand how NPs modulate bond-exchange kinetics, their role must be considered not only from a chemical perspective but also through the classical polymer physics of the filler–matrix interphase. In conventional polymer nanocomposites composed of a permanently cross-linked polymer matrix, the interphase governs stress transfer and reinforcement through covalent bonding and/or supramolecular interactions, such as van der Waals forces, dipolar interactions, and hydrogen bonding. These interactions restrict segmental mobility and can result in the formation of a bound-polymer layer with local properties that differ from the bulk matrix, such as altered morphology, thermal behavior, dielectric response, and viscoelastic dynamics.^101^

In terms of stress relaxation, unmodified fillers already promote a temperature-dependent change in viscoelastic properties in conventional polymer nanocomposites by physical processes such as reptation.^102^ In dynamic polymer nanocomposites, this interphase becomes an active region where chain mobility and bond-exchange kinetics are coupled. Unmodified fillers can act as physical obstacles to chain rearrangement and bond reshuffling, particularly when the bond exchange becomes the dominant relaxation mechanism. As a result, reduced segmental mobility near the filler surface may slow the apparent macroscopic stress relaxation and suppress viscoelastic flow compared to the unfilled dynamic network. Such behavior has been reported for dynamic nanocomposites containing unmodified inorganic particles such as silica and alumina.^76,103,104,105^

Surface modification offers a route to transform this otherwise restrictive interphase into a region that promotes the exchange. Covalent bonding of the matrix to the filler surface and/or the introduction of exchangeable or catalytic groups increase the local concentration of reactive functionalities, which is especially effective for nanosized fillers because of their high specific surface area. In this case, bond exchange can be promoted in the immediate vicinity of the particles, enabling stress relaxation of chain segments by chemically induced network rearrangement. However, the relaxation behavior of dynamic nanocomposites is not governed by functional-group density alone but by the coupled effects of interfacial chemistry, segmental mobility, particle type, loading, orientation, and dispersion.^76^

Importantly, NP surface modification should not be interpreted as producing a clean or spatially uniform change in the apparent *E*_a_. Instead, it may create a spatially heterogeneous kinetic environment across the filler–matrix interphase, in which segmental α-relaxation and bond-exchange-mediated relaxation are affected differently. Polymer chains directly bound to, or strongly adsorbed near, the NPs surface experience restricted mobility and slower α-relaxation than the bulk matrix, whereas surface-bound catalytic or exchangeable groups can locally accelerate bond exchange.^106,107^ Thus, interfacial stress relaxation is governed by the competition between a mobility-limited process, controlled by segmental dynamics, and a chemistry-limited process, controlled by the rate of bond exchange. This interpretation is consistent with transient-network descriptions of vitrimer nanocomposites, in which nanofillers reduce the mobility of the dynamic network and increase the effective rigid/static network fraction.^108^ Consequently, the macroscopic relaxation response reflects the balance between interfacial confinement and catalytic acceleration rather than surface chemistry alone.^106,107^

This competition between segmental mobility and bond-exchange kinetics is particularly relevant for semicrystalline polyester-based polymers such as PCL.^109^ In PCL nanocomposites, the low melting point of the matrix, crystalline phase, and reduced chain mobility near the filler surface can all overlap with chemically induced network flow.^81–83^ Although the low *T*_g_ of PCL provides high segmental mobility in the bulk matrix, polymer chains located near inorganic nanoparticle surfaces may form a confined interfacial layer in which mobility is locally retarded by adsorption, hydrogen bonding, or physical confinement. Thus, even when surface-bound catalytic groups accelerate local bond exchange, the overall relaxation response may remain limited by slowed segmental dynamics at the filler–matrix interphase.^109^ Therefore, lowering apparent *T*_v_ toward mildly thermally stimulated biomedical operating conditions may require more than catalytic surface chemistry alone; the interphase should ideally promote bond exchange while preserving sufficient local segmental mobility. Possible strategies include immobilizing catalytic groups, such as Brønsted acids or bases, on the nanoparticle surface,^110–112^ or grafting flexible polymer brushes that create a more mobile interphase.^113^ Thermoplastic organic particles with low thermal transitions may provide an additional route to enhance interfacial mobility and accelerate exchange under mild conditions.^114,115^ These considerations highlight that apparent *E*_a_ and *T*_v_ in dynamic polymer nanocomposites are not intrinsic properties of the polymer backbone alone, but emergent parameters governed by the local chemical and physical environment of the filler–matrix interphase.

### 4.3 Dynamic PCL-Based Nanocomposites: Limited but Critical Evidence

The integration of NPs into PCL-based dynamic polymers remains underexplored, with only a few studies reported to date (Table 4). Existing work combines dynamic covalent PCL networks with nanoscale fillers to improve mechanical performance, recyclability, and stress relaxation behavior. However, a systematic understanding of how NP–dynamic polymer interfaces influence *E*_a_ and *T*_v_ in dynamic PCL networks is still largely missing, representing a critical gap in the field.

**Table 4:** Representative PCL-Based and PCL-Containing Dynamic Polymers Relevant to Nanocomposite Design

Network	Nanofiller	Chemistry	Catalyst	Apparent *E*_a_ (kJ/mol)	Apparent *T*_v_/Relaxation	Key Insight	Ref
PCL (DBEDT)	No filler	Boronic ester exchange	None	44–62	Not reported	Fully biodegradable vitrimer	^14^
PCL + rGO	rGO	Transesterification/transcarbamoylation	Sn(Oct)_2_	Not reported	Not reported	Reinforcement without kinetic data	^116^
PCL + MWCNT	MWCNT	Furan/maleimide Diels–Alder	None	Not reported	Not reported	MWCNT-enabled Joule heating	^117^
Epoxy–thiol + CNC–PCL	CNC–PCL	Transesterification	Network-driven	Not reported	Tunable relaxation	Interfacial grafting controls dynamics	^113^

From a chemical perspective, PCL-based dynamic polymers have been developed using several associative exchange mechanisms. A representative example is the biodegradable network reported by Damonte et al., where star-shaped acrylated PCL is cross-linked with a diboronic ester dithiol (DBEDT). The resulting network undergoes boronic ester exchange and exhibits activation energies in the range 44–62 kJ mol^–1^, while maintaining full enzymatic degradability within 12 days. Notably, no external catalyst is required, as exchange reactions are intrinsic to the boronic ester chemistry. However, this PCL dynamic polymer does not incorporate NPs and therefore reflects predominantly bulk-controlled bond-exchange kinetics.^14^

In contrast, PCL-based associative networks incorporating NPs rely on catalytically activated exchange mechanisms. Vallin et al. reported a PCL-based dynamic covalent network filled with reduced graphite oxide (rGO), where hydroxyl-terminated PCL is cross-linked via urethane bonds capable of transesterification and transcarbamoylation. In this polymer, Sn­(Oct)_2_ enables dynamic bond exchange. The incorporation of rGO enhances melt viscosity, promotes solid-like rheological behavior, and preserves self-healing and recyclability. However, quantitative data on *E*_a_ and *T*_v_ are not reported, and the role of NP–dynamic polymer interfaces in controlling exchange kinetics remains unresolved.^116^

A related interfacial design strategy is demonstrated in dynamic epoxy–thiol nanocomposites incorporating PCL-grafted cellulose nanocrystals (CNC–PCL). In this dynamic network, cellulose nanocrystals functionalized with PCL chains form core–shell nanostructures that improve compatibility with the matrix and enable covalent integration into the dynamic network through transesterification. Nanofiller loading (0–20 wt %) significantly influences both mechanical and dynamic properties. At approximately 15 wt %, the material exhibits increase of 2.5× in Young’s modulus, 5.4× in fracture stress, and 2.0× in fracture strain. Importantly, the nanofiller also modifies stress relaxation behavior, indicating that interfacial interactions influence exchange kinetics and effective *E*_a_, although explicit *E*_a_ and *T*_v_ values are not reported.^113^

Taken together, these studies highlight that dynamic PCL-based nanocomposites remain at an early stage of development, with only isolated examples demonstrating partial integration of dynamic chemistry and interfacial effects. In particular, none of the reported PCL-based dynamic nanocomposites provide a direct, quantitative link between NP surface chemistry and bond-exchange kinetics (*E*_a_, *T*_v_), despite clear indications that such effects are present. This underscores the need for design strategies capable of directly probing and controlling interfacial contributions to bond-exchange kinetics.

This limited body of evidence highlights a critical gap: the absence of material platform that integrates PCL chemistry, dynamic covalent exchange, and NP-enabled modulation of *E*_a_ and *T*_v_ within a unified and predictive framework. Insights from broader dynamic nanocomposite research indicate that nanoparticles may alter exchange-mediated relaxation and apparent *T*_v_ through interfacial chemical and physical effecta.^16,118^ These findings suggest that NP–dynamic polymer interfaces can act as local kinetic modifiers, yet this concept has not been systematically investigated or quantitatively linked to the apparent *E*_a_, kinetic prefactor, τ, and *T*_v_ of dynamic PCL-based networks.

### 4.4 Emerging Understanding: Nanoparticles as Kinetic Modulators

In dynamic nanocomposites, NPs should not be considered solely as passive reinforcing agents, but rather as active modifiers of network dynamics. Their influence extends beyond mechanical performance into the kinetic domain, where the apparent *E*_a_, kinetic prefactor, τ, and apparent *T*_v_, can be strongly influenced by NP–dynamic polymer interfaces.

Depending on their surface chemistry and interaction with the surrounding matrix, NPs may reduce *E*_a_ and accelerate stress relaxation, particularly when their surfaces exhibit catalytic activity or high polarity that promotes bond-exchange reactions.^71,88^ In addition, certain NPs enable localized activation mechanisms, such as photothermal or magnetic heating, allowing spatially controlled bond exchange without globally heating the entire material.^16^

These effects are not intrinsic to the NPs alone, but they are governed by dispersion, loading, and most critically, surface functionalization. Interfacial chemistry defines the accessibility and reactivity of dynamic bonds, effectively shaping the local kinetic environment of bond exchange. As a result, NP–dynamic polymer interfaces act as localized kinetic environments within otherwise homogeneous networks.^16^

A clear gap emerges from the current literature. PCL-based dynamic polymers exhibit bond exchange but remain governed by bulk kinetics. Conventional PCL nanocomposites display strong interfacial effects but lack dynamic exchange. Meanwhile, dynamic nanocomposites in other dynamic polymer materials demonstrate interfacial control over *E*_a_ and *T*_v_ but are typically based on nonbiomedical matrices such as epoxies.^71,78,81,84,86,90,95,96,119–123^

To the best of our knowledge, no reported material has yet systematically combined a biocompatible PCL matrix, dynamic covalent chemistry, and NP-enabled modulation of exchange kinetics within a single, unified dynamic material.

Taken together, these observations demonstrate that the apparent *E*_a_, τ, and apparent *T*_v_ are not intrinsic properties of the PCL backbone, but rather emergent parameters governed by exchange chemistry, polymer dynamics, and the local chemical and physical environment. However, current strategies remain largely bulk-centric, resulting in spatially homogeneous dynamics and limiting the ability to achieve localized or programmable control. This limitation becomes particularly critical in advanced applications such as self-healing biomaterials, adaptive implants, and stimuli-responsive systems, where selective activation of exchange reactions is essential.

## 5 Future Perspectives: Interfacial Engineering Strategies for Programming Bond-Exchange Dynamics

While conventional design of dynamic networks relies on bulk parameters such as catalyst concentration, cross-link density, and polymer architecture, these approaches inherently treat apparent *E*_a_ and *T*_v_ as uniform, global properties.^4,50^ In contrast, growing evidence from dynamic nanocomposites demonstrates that NP–dynamic polymer interfaces can significantly influence exchange kinetics, stress relaxation, and viscoelastic response by creating localized chemical environments.^16^

Building on this understanding, the behavior of dynamic polymers can be reframed through an interfacial kinetic engineering approach in which NP surfaces act as localized regulators of dynamic covalent exchange. This concept is particularly relevant for PCL-based dynamic polymers, where the high intrinsic chain mobility may amplify the effect of changes in *E*_a_, potentially resulting in pronounced shifts in the *T*_v_. One strategy involves introducing surface functionalities that are capable of participating in or catalyzing exchange reactions. NPs functionalized with hydroxyl, amine, or basic groups can locally enhance nucleophilicity, facilitate transesterification, and potentially accelerate exchange reactions in the interfacial region. In this way, NP surfaces act as confined catalytic domains, creating spatially heterogeneous kinetics where exchange reactions proceed faster at interfaces than in the bulk matrix.

Interfacial polarity and chemical compatibility govern the segmental mobility and accessibility of reactive groups. Interfaces that are chemically compatible with the PCL matrix promote chain mobility and facilitate exchange reactions, whereas poorly compatible or hydrophobic surfaces suppress interfacial interactions and limit their kinetic contributions. Conversely, strong hydrogen-bonding interactions or rigid interfaces can immobilize polymer chains, increasing *E*_a_ and shifting *T*_v_ toward higher temperatures. This establishes a bidirectional design principle in which nanoparticle interfaces can either accelerate or suppress dynamic exchange depending on their chemical functionality.

Another key parameter is the interfacial volume fraction, which is determined by nanoparticle size, dispersion, and loading. NPs with high surface area increase the fraction of interfacial regions and amplify their influence on macroscopic behavior, whereas aggregation reduces effective interfacial contributions. Achieving uniform dispersion is therefore essential for translating nanoscale interactions into a predictable dynamic network performance.

In this context, the interfacial volume fraction should be linked to percolation. At low nanoparticle loadings, dynamic interfacial zones remain isolated and mainly affect the local bond-exchange kinetics. With increasing filler concentration or effective interphase thickness, for example, through adsorption, surface grafting, or polymer brush formation, neighboring interfacial zones can overlap and form a percolated dynamic interfacial network,^124,125^ Above this threshold, the interface is no longer a localized kinetic modifier but can dominate the bulk viscoelastic response,^125,126^ including stress relaxation, flow, and apparent *T*_v_. The percolation threshold is system-specific and depends on particle size, aspect ratio, dispersion, aggregation, interphase thickness, and surface chemistry; high-aspect-ratio fillers such as graphene or carbon nanotubes are therefore expected to percolate at lower loadings than spherical particles.^126–128^

A practical formulation strategy should therefore optimize catalytic or coordination-group density and NP loading together, rather than maximizing either parameter independently. Excessively high grafting density may enchance local exchange activity but may also restrict nearby chain mobility, promote particle association, and enhance background creep if interfacial exchange becomes too extensive.^129,130^ Conversely, too low a grafting density may preserve mobility but provide insufficient kinetic modulation. An effective approach is to use high-surface-area or high-aspect-ratio NPs at low-to-moderate loadings, combined with moderate grafting densities and flexible spacer or brush architectures that maintain interfacial mobility and dispersion.^128,129^ Formulations should be screened by independently varying particle loading, for .example within 1.0–10.0 wt %, and surface functional-group density, followed by evaluation of *T*_v_ or τ­(*T*), creep below the activation window, stress relaxation under mild stimulation, and dispersion quality. The target is not the maximum exchange rate, but selective activation under stimulation while maintaining dimensional stability under baseline conditions.^127^

Despite their potential, several challenges remain. Achieving uniform nanoparticle dispersion, maintaining stable and well-defined interfacial chemistry during processing, and decoupling interfacial effects from bulk contributions are nontrivial. Furthermore, the long-term stability and potential leaching of functionalized nanoparticles must be carefully evaluated, particularly in biomedical applications.

Future research should therefore focus on establishing quantitative relationships among interfacial chemistry, *E*_a_, and macroscopic response. The development of experimental methodologies capable of probing localized exchange kinetics, combined with multiscale modeling approaches, will be essential for translating interfacial design principles into predictive material frameworks.

Advanced characterization methods will be essential to verify whether the proposed interfacial kinetic gradients are physically realized. Broadband dielectric spectroscopy (BDS) can resolve segmental α-relaxation and, where sufficient dielectric contrast and frequency separation exist, distinguish it from slower ionic, network-strand, or exchange-related processes.^35^ In parallel, AIE-based fluorescence probes or fluorescence microscopy could be used to report changes in local mobility and packing that may be associated with topology rearrangement and identify spatially heterogeneous dynamic regions near nanoparticle-rich domains.^22^ Rheo-NMR or solid-state NMR under deformation may further provide molecular-level information on chain orientation, segmental mobility, and exchange-driven network rearrangement under mechanical or thermal stimulation.^132,133^ Combining these methods with conventional rheology, DSC, and structural techniques such as small/wide-angle X-ray scattering (SAXS/WAXS) would enable separation of crystallinity-controlled relaxation, bulk viscoelasticity, and truly interfacial bond-exchange dynamics.^27^

Recent advances in molecular simulation provide a complementary pathway for testing nanoscale interfacial kinetic gradients. Reactive molecular dynamics using force fields such as ReaxFF, accelerated molecular dynamics, hybrid MD–Monte Carlo approaches, and coarse-grained vitrimer models have been used to simulate bond exchange, stress relaxation, healing, and rheological behavior in covalent adaptable networks and vitrimer nanocomposites. For functionalized NPs, such approaches could map bond-exchange probability, local relaxation times, and apparent activation barriers as a function of distance from the particle surface. Atomistic or reactive simulations could probe how catalytic or coordination groups modify local reaction pathways, whereas coarse-grained models could capture interphase thickness, filler loading, aspect ratio, and percolation of dynamic interfacial zones. Such a multiscale workflow would link surface chemistry, local *E*_a_ gradients, segmental mobility, and macroscopic relaxation behavior.^134–137^

Overall, interfacial engineering represents a fundamental shift from bulk-controlled materials to spatially programmable dynamic networks.

By treating NP–polymer interfaces as active kinetic regulators, local exchange and relaxation rates may be modulated, thereby altering the bulk apparent *E*_a_ and *T*_v_ or enabling spatially selective responses under external stimulation.

## Abbreviations

PCL: poly­(ε-caprolactone)
CAN: covalent adaptable network
NPs: nanoparticles
*T*_v_: topology-freezing temperature
*T*_g_: glass transition temperature
*E*_a_: activation energy
τ: relaxation time
DMA: dynamic mechanical analysis
rGO: reduced graphene oxide
GO: graphene oxide
CNT: carbon nanotube
CNC: cellulose nanocrystal
CNF: cellulose nanofibril
SiO_2_: silicon dioxide
Fe_3_O_4_: magnetite
Sn­(Oct)_2_: tin­(II) 2-ethylhexanoate
DBTDL: dibutyltin dilaurate
TBD: 1,5,7-triazabicyclo­[4.4.0]­dec-5-ene
PPh_3_: triphenylphosphine
Zn­(OAc)_2_: zinc acetate
DBEDT: diboronic ester dithiol
DSC: differential scanning calorimetry
SAXS: small-angle X-ray scattering
WAXS: wide-angle X-ray scattering
BDS: Broadband Dielectric Spectroscopy
AIE: aggregation-induced emission

## References

[1] Miao W., Zou W., Luo Y., Zheng N., Zhao Q., Xie T. (2020). Structural
tuning of polycaprolactone based thermadapt shape memory polymer. Polym. Chem..

[2] Lv J., Wang A., Li Y., Xu S., Li X. (2023). Thermadapt
Shape Memory PCL- *co* -PBA Network Exhibiting Tunable
Properties and Dynamics of Ester–Ester Exchange Reactions. ACS Appl. Polym. Mater..

[3] Li X., Wang L., Li Y., Xu S. (2023). Reprocessable, Self-Healing,
Thermadapt Shape Memory Polycaprolactone via Robust Ester–Ester
Interchanges Toward Kirigami-Tailored 4D Medical Devices. ACS Appl. Polym. Mater..

[4] Van
Zee N. J., Nicolaÿ R. (2020). Vitrimers: Permanently crosslinked
polymers with dynamic network topology. Prog.
Polym. Sci..

[5] Montarnal D., Capelot M., Tournilhac F., Leibler L. (2011). Silica-Like Malleable
Materials from Permanent Organic Networks. Science
(1979).

[6] Capelot M., Montarnal D., Tournilhac F., Leibler L. (2012). Metal-Catalyzed Transesterification
for Healing and Assembling of Thermosets. J.
Am. Chem. Soc..

[7] Klingler A., Reisinger D., Schlögl S., Wetzel B., Breuer U., Krüger J.-K. (2024). Vitrimer
Transition Phenomena from the Perspective
of Thermal Volume Expansion and Shape (In)­stability. Macromolecules.

[8] Invernizzi M., Turri S., Levi M., Suriano R. (2018). 4D printed thermally
activated self-healing and shape memory polycaprolactone-based polymers. Eur. Polym. J..

[9] Xu, J. ; Song, J. Thermal Responsive Shape Memory Polymers for Biomedical Applications. Biomedical Engineering - Frontiers and Challenges; InTech, 2011; 10.5772/19256.

[10] Pinto L. A. (2024). Shape memory thermoplastic polyurethane/polycaprolactone blend and
composite with hydroxyapatite for biomedical application. J. Mater. Res..

[11] Joe J. (2021). A 4D Printable Shape
Memory Vitrimer with Repairability and Recyclability
through Network Architecture Tailoring from Commercial Poly­(*ε* -caprolactone). Advanced Science.

[12] Zhang L., Qiu T., Zhu Z., Guo L., Li X. (2018). Self-Healing Polycaprolactone
Networks through Thermo-Induced Reversible Disulfide Bond Formation. Macromol. Rapid Commun..

[13] Ghosh S. (2025). Towards cell-adhesive, 4D printable PCL networks through dynamic
covalent chemistry. J. Mater. Chem. B.

[14] Damonte G. (2024). On Sustainable Vitrimers
Based on Polycaprolactones. ACS Appl. Polym.
Mater..

[15] Bischoff D. J., Mackay M. E. (2024). Plasticization of a Diels–Alder Covalent Adaptable
Network for Fused Filament Fabrication. ACS
Appl. Polym. Mater..

[16] Ghosh S. (2025). Impact of nanofillers
on vitrimerization and recycling strategies:
a review. Nanoscale Adv..

[17] Wang S. (2023). Nanotechnology in Covalent
Adaptable Networks: from Nanocomposites
to Surface Patterning. ACS Mater. Lett..

[18] Hubbard A. M. (2022). Vitrimer Composites: Understanding the Role of Filler in Vitrimer
Applicability. ACS Appl. Polym. Mater..

[19] Yang H., Hu K., Wang D. (2022). Using Nanosphere Embedding to Probe the Surface and
Bulk Relaxation in Vitrimers. Langmuir.

[20] Meng F., Saed M. O., Terentjev E. M. (2022). Rheology of vitrimers. Nat. Commun..

[21] Hubbard A. M. (2021). Vitrimer Transition Temperature Identification:
Coupling Various
Thermomechanical Methodologies. ACS Appl. Polym.
Mater..

[22] Yang Y., Zhang S., Zhang X., Gao L., Wei Y., Ji Y. (2019). Detecting topology freezing transition temperature of vitrimers by
AIE luminogens. Nat. Commun..

[23] Nishimura Y., Chung J., Muradyan H., Guan Z. (2017). Silyl Ether as a Robust
and Thermally Stable Dynamic Covalent Motif for Malleable Polymer
Design. J. Am. Chem. Soc..

[24] Lee G., Song H. Y., Choi S., Bin Kim C., Hyun K., Ahn S. (2022). Harnessing β-Hydroxyl
Groups in Poly­(β-Amino Esters)
toward Robust and Fast Reprocessing Covalent Adaptable Networks. Macromolecules.

[25] Perego A., Khabaz F. (2023). Creep and Recovery
Behavior of Vitrimers with Fast
Bond Exchange Rate. Macromol. Rapid Commun..

[26] Fang H. (2023). Rheology of Smectic
Liquid Crystalline Elastomers with Dynamic Covalent
Bonds. Macromolecules.

[27] Hayashi M., Suzuki M., Kito T. (2025). Understanding
the Topology Freezing
Temperature of Vitrimer-Like Materials through Complementary Structural
and Rheological Analyses for Phase-Separated Network. ACS Macro Lett..

[28] Denissen W., Winne J. M., Du Prez F. E. (2016). Vitrimers:
permanent organic networks
with glass-like fluidity. Chem. Sci..

[29] Carden G.
P. (2024). Critical
Role of Free Amine Groups in the Imine Bonds Exchange in
Dynamic Covalent Networks. Macromolecules.

[30] Ricarte R. G., Shanbhag S. (2021). Unentangled Vitrimer Melts: Interplay between Chain
Relaxation and Cross-link Exchange Controls Linear Rheology. Macromolecules.

[31] Xia J., Kalow J. A., Olvera
de la Cruz M. (2023). Structure, Dynamics, and Rheology
of Vitrimers. Macromolecules.

[32] Lin T.-W., Mei B., Dutta S., Schweizer K. S., Sing C. E. (2025). Molecular Dynamics
Simulation and Theoretical Analysis of Structural Relaxation, Bond
Exchange Dynamics, and Glass Transition in Vitrimers. Macromolecules.

[33] Carden G. P. (2025). Critical Role of the
Steric Factor in the Viscoelasticity of Vitrimers. Macromolecules.

[34] Röttger M., Domenech T., van der
Weegen R., Breuillac A., Nicolaÿ R., Leibler L. (2017). High-performance vitrimers from commodity
thermoplastics through dioxaborolane metathesis. Science (1979).

[35] Soman B., Schweizer K. S., Evans C. M. (2023). Fragile Glass Formation and Non-Arrhenius
Upturns in Ethylene Vitrimers Revealed by Dielectric Spectroscopy. Macromolecules.

[36] Cui X., Jiang N., Shao J., Zhang H., Yang Y., Tang P. (2023). Linear and Nonlinear
Viscoelasticities of Dissociative and Associative
Covalent Adaptable Networks: Discrepancies and Limits. Macromolecules.

[37] Barzycki D. C., Ezzeddine D., Shanbhag S., Ricarte R. G. (2025). Linear Viscoelasticity
of Polystyrene Vitrimers: Segmental Motions and the Slow Arrhenius
Process. Macromolecules.

[38] Lin T.-W., Mei B., Dutta S., Schweizer K. S., Sing C. E. (2025). Molecular Dynamics
Simulation and Theoretical Analysis of Structural Relaxation, Bond
Exchange Dynamics, and Glass Transition in Vitrimers. Macromolecules.

[39] Ge S., Evans C. M. (2025). Influence of Segmental
Dynamics on Bond Exchange in
Imine Vitrimers with Different Polymer Backbones and Cross-Linkers. Macromolecules.

[40] Majumdar S. (2021). Efficient Exchange in
a Bioinspired Dynamic Covalent Polymer Network
via a Cyclic Phosphate Triester Intermediate. Macromolecules.

[41] Schäler K., Achilles A., Bärenwald R., Hackel C., Saalwächter K. (2013). Dynamics in
Crystallites of Poly­(ε-caprolactone) As Investigated by Solid-State
NMR. Macromolecules.

[42] Harrison K. L., Jenkins M. J. (2004). The effect of crystallinity
and water absorption on
the dynamic mechanical relaxation behaviour of polycaprolactone. Polym. Int..

[43] Ferry, J. D. Viscoelastic properties of polymers; Wiley, 1980.

[44] Münstedt H. (2014). Rheological
experiments at constant stress as efficient method to characterize
polymeric materials. J. Rheol. (N. Y. N. Y)..

[45] Ricarte R.
G., Shanbhag S. (2024). A tutorial
review of linear rheology for polymer chemists:
basics and best practices for covalent adaptable networks. Polym. Chem..

[46] Hubbard A. M., Ren Y., Sarvestani A., Picu C. R., Varshney V., Nepal D. (2023). Thermomechanical
analysis (TMA) of vitrimers. Polym. Test..

[47] Kaiser S. (2020). “The crucial
role of external force in the estimation of the
topology freezing transition temperature of vitrimers by elongational
creep measurements,”. Polymer (Guildf).

[48] Klingler A., Reisinger D., Schlögl S., Wetzel B., Breuer U., Krüger J.-K. (2024). Vitrimer
Transition Phenomena from the Perspective
of Thermal Volume Expansion and Shape (In)­stability. Macromolecules.

[49] Wang Y. (2025). Ellipsometric Characterization
of Network Topology Transition in
Vitrimers. ACS Macro Lett..

[50] Capelot M., Unterlass M. M., Tournilhac F., Leibler L. (2012). Catalytic Control of
the Vitrimer Glass Transition. ACS Macro Lett..

[51] Guerre M., Taplan C., Winne J. M., Du Prez F. E. (2020). Vitrimers: directing
chemical reactivity to control material properties. Chem. Sci..

[52] Bhusal S. (2021). Transesterification
in Vitrimer Polymers Using Bifunctional Catalysts:
Modeled with Solution-Phase Experimental Rates and Theoretical Analysis
of Efficiency and Mechanisms. J. Phys. Chem.
B.

[53] Niu X., Wang F., Li X., Zhang R., Wu Q., Sun P. (2019). Using Zn ^2+^ Ionomer To Catalyze Transesterification Reaction
in Epoxy Vitrimer. Ind. Eng. Chem. Res..

[54] Tanzi M. C., Verderio P., Lampugnani M. G., Resnati M., Dejana E., Sturani E. (1994). Cytotoxicity of some catalysts commonly used in the
synthesis of copolymers for biomedical use. J. Mater. Sci. Mater. Med..

[55] Self J. L., Dolinski N. D., Zayas M. S., Read de Alaniz J., Bates C. M. (2018). Brønsted-Acid-Catalyzed Exchange
in Polyester
Dynamic Covalent Networks. ACS Macro Lett..

[56] Gadomska-Gajadhur A., Ruśkowski P. (2020). Biocompatible Catalysts for Lactide Polymerization—Catalyst
Activity, Racemization Effect, and Optimization of the Polymerization
Based On Design of Experiments. Org. Process
Res. Dev..

[57] Majumdar S. (2021). Efficient Exchange in a Bioinspired Dynamic
Covalent Polymer Network
via a Cyclic Phosphate Triester Intermediate. Macromolecules.

[58] Stjerndahl A., Finne-Wistrand A., Albertsson A.-C., Bäckesjö C. M., Lindgren U. (2008). Minimization
of residual tin in the controlled Sn­(II)­octoate-catalyzed
polymerization of ε-caprolactone. J. Biomed.
Mater. Res., Part A.

[59] Elling B. R., Dichtel W. R. (2020). Reprocessable Cross-Linked
Polymer Networks: Are Associative
Exchange Mechanisms Desirable?. ACS Cent. Sci..

[60] Majumdar S. (2021). Efficient Exchange in a Bioinspired Dynamic Covalent Polymer Network
via a Cyclic Phosphate Triester Intermediate. Macromolecules.

[61] Chien S.-Y., Wang J., Liu Y.-L. (2024). Biodegradable
Polyester-Based Vitrimers
Exhibiting Transesterification-Induced Topography Isomerization under
Recycling. ACS Appl. Polym. Mater..

[62] Gosecki M., Gosecka M. (2022). Boronic Acid Esters and Anhydrates as Dynamic Cross-Links
in Vitrimers. Polymers (Basel).

[63] Zhang X., Wang S., Jiang Z., Li Y., Jing X. (2020). Boronic Ester
Based Vitrimers with Enhanced Stability via Internal Boron–Nitrogen
Coordination. J. Am. Chem. Soc..

[64] Dhers S., Vantomme G., Avérous L. (2019). A fully bio-based polyimine vitrimer
derived from fructose. Green Chem..

[65] Li X., Wu T. (2021). Rheological and mechanical
properties of dynamic covalent polymers
based on imine bond. J. Appl. Polym. Sci..

[66] Winne J. M., Leibler L., Du Prez F. E. (2019). Dynamic
covalent chemistry in polymer
networks: a mechanistic perspective. Polym.
Chem..

[67] Zhang V., Kang B., Accardo J. V., Kalow J. A. (2022). Structure–Reactivity–Property
Relationships in Covalent Adaptable Networks. J. Am. Chem. Soc..

[68] Konuray O., Fernández-Francos X., Ramis X. (2023). Structural Design of
CANs with Fine-Tunable Relaxation Properties: A Theoretical Framework
Based on Network Structure and Kinetics Modeling. Macromolecules.

[69] Wang H., Guo S., Zhang X., Liu Y., Liu T., Yu H. (2022). Insight into
the structure-property relationships of intramolecularly-catalyzed
epoxy vitrimers. Mater. Des..

[70] Cuminet F., Caillol S., Dantras É., Leclerc É., Ladmiral V. (2021). Neighboring
Group Participation and Internal Catalysis Effects on Exchangeable
Covalent Bonds: Application to the Thriving Field of Vitrimer Chemistry. Macromolecules.

[71] Yue L., Amirkhosravi M., Ke K., Gray T. G., Manas-Zloczower I. (2021). Cellulose
Nanocrystals: Accelerator and Reinforcing Filler for Epoxy Vitrimerization. ACS Appl. Mater. Interfaces.

[72] Sun M. (2026). Covalent Adaptable Networks:
Reprocessable Cross-Linked Polymers. Chem. Rev..

[73] Hayashi M., Ricarte R. G. (2025). Towards the next development of vitrimers: Recent key
topics for the practical application and understanding of the fundamental
physics. Prog. Polym. Sci..

[74] Liu Y., Hsu S. (2018). Synthesis and Biomedical
Applications of Self-healing Hydrogels. Front.
Chem..

[75] Kaur G., Kumar P., Sonne C. (2024). Synthesis,
properties and biomedical
perspective on vitrimers – challenges & opportunities. RSC Applied Interfaces.

[76] Legrand A., Soulié-Ziakovic C. (2016). Silica–Epoxy
Vitrimer Nanocomposites. Macromolecules.

[77] Kausar A., Ahmad I. (2024). State-of-the-art epoxy vitrimer nanocomposites with graphene, carbon
nanotube and silica—fundamentals and applications (radiation
shielding, sensing and corrosion resistance). Journal of Plastic Film & Sheeting.

[78] Li Z.-X., Xiang Y.-S., Ran Y. (2023). Well-dispersed
silica nanoparticle-reinforced
epoxy vitrimer composites prepared by dynamic crosslinking. J. Mater. Sci..

[79] Dey I., Kundu D., Ghosh S., Mandal S., Samanta K., Bose S. (2025). Vitrimer nanocomposites’
derived from graphene oxide and post-consumer
recycled polypropylene. Nanoscale Adv..

[80] Ren Y. (2024). Rapid Photothermal Healing
of Vitrimer Nanocomposites Activated by
Gold-Nanoparticle-Coated Graphene Nanoplatelets. ACS Appl. Nano Mater..

[81] Caglayan C., Kim G., Yun G. J. (2022). CNT-Reinforced
Self-Healable Epoxy Dynamic Networks
Based on Disulfide Bond Exchange. ACS Omega.

[82] Feng Y. (2022). An Effective Approach to Improve the Thermal Conductivity, Strength,
and Stress Relaxation of Carbon Nanotubes/Epoxy Composites Based on
Vitrimer Chemistry. Int. J. Mol. Sci..

[83] Lorwanishpaisarn N. (2022). Self-healing Ability
of Epoxy Vitrimer Nanocomposites Containing
Bio-Based Curing Agents and Carbon Nanotubes for Corrosion Protection. J. Polym. Environ..

[84] Bohra B. S. (2023). “Specific functionalized
graphene oxide-based vitrimer epoxy
nanocomposites for self-healing applications,”. Compos. Sci. Technol..

[85] Shen S., Kinloch I. A., Vallés C. (2025). “Role of the aspect ratio
of graphene oxide (GO) on the interface and mechanical properties
of vitrimer/GO nanocomposites,”. Compos.
Sci. Technol..

[86] Zhang S. (2026). Nanocomposites based on epoxy vitrimer. Nano
Res..

[87] Zengin A., Castro J. P. O., Habibovic P., van Rijt S. H. (2021). Injectable, self-healing
mesoporous silica nanocomposite hydrogels with improved mechanical
properties. Nanoscale.

[88] Huang Z., Wang Y., Zhu J., Yu J., Hu Z. (2018). Surface engineering
of nanosilica for vitrimer composites. Compos.
Sci. Technol..

[89] Poutrel Q.-A., Baghdadi Y., Souvignet A., Gresil M. (2021). Graphene functionalisations:
Conserving vitrimer properties towards nanoparticles recovery using
mild dissolution. Compos. Sci. Technol..

[90] Yang Y., Pei Z., Zhang X., Tao L., Wei Y., Ji Y. (2014). Carbon nanotube–vitrimer
composite for facile and efficient photo-welding of epoxy. Chem. Sci..

[91] Diodati L. E., Liu S., Rinaldi-Ramos C. M., Sumerlin B. S. (2023). Magnetic Nanoparticles
Improve Flow Rate and Enable Self-Healing in Covalent Adaptable Networks. ACS Appl. Mater. Interfaces.

[92] Kaiser S., Jandl J., Novak P., Schlögl S. (2020). Design and
characterisation of vitrimer-like elastomeric composites from HXNBR
rubber. Soft Matter.

[93] Wu Y., Wei Y., Ji Y. (2023). Carbon material/vitrimer
composites: Towards sustainable,
functional, and high-performance crosslinked polymeric materials. Giant.

[94] Chen J. (2019). Vitrimer Chemistry Assisted
Fabrication of Aligned, Healable, and
Recyclable Graphene/Epoxy Composites. Front.
Chem..

[95] Wu J. (2026). Functional Carbon Nanotubes
Reinforced Epoxy Vitrimer Nanocomposites
for Recyclable Strain Sensors. Polym. Composites.

[96] Ran Y., Li Y.-D., Zeng J.-B. (2022). “Dynamic
crosslinking towards
well-dispersed cellulose nanofiber reinforced epoxy vitrimer composites,”. Composites Communications.

[97] Gao M. (2022). Design and fabrication of recyclable and reshape vitrified
elastomer
reinforced with renewable cellulose nanocrystal. Composites Communications.

[98] Castro-Cabrera I., Fahs A., Berlioz S., Carriere P. (2026). Optimizing Epoxy Vitrimer
Composites With Sustainable Cellulose Nanofibril Reinforcements: Enhancing
Thermal Dimensional Stability While Preserving Dynamic Covalent Behavior. Polym. Compos..

[99] Wang Y., Gao M., Li S., Liu J., Feng A., Zhang L. (2022). Recyclable,
self-healable and reshape vitrified poly-dimethylsiloxane composite
filled with renewable cellulose nanocrystal. Polymer (Guildf)..

[100] Signorato G., Klauke L. R., Haida P., Vossmeyer T., Abetz V. (2025). Superparamagnetic iron oxide nanoparticle – vitrimer nanocomposites:
Reprocessable and multi-responsive materials. Polymer (Guildf)..

[101] Khan, I. ; Polymer nanocomposites: an overview. Smart Polymer Nanocomposites; Elsevier, 2023; pp 167–184, 10.1016/B978-0-323-91611-0.00017-7.

[102] de Gennes P. G. (1971). Reptation
of a Polymer Chain in the Presence of Fixed
Obstacles. J. Chem. Phys..

[103] Breuer, U. ; Wetzel, B. ; Gurka, M. Multifunctionality of polymer composites: challenges and applications; William Andrew, 2025.

[104] Chen Z., Li Z., Wang X., Jin C., Ning D. (2024). Strengthening and toughening of hard epoxy vitrimer
nanocomposites
with interfacial covalent binding and microphase separation. Compos. Sci. Technol..

[105] Lossada F., Jiao D., Hoenders D., Walther A. (2021). Recyclable
and Light-Adaptive Vitrimer-Based Nacre-Mimetic Nanocomposites. ACS Nano.

[106] Cheng S. (2015). Revealing spatially
heterogeneous relaxation in a model
nanocomposite. J. Chem. Phys..

[107] Holt A. P. (2016). Controlling Interfacial
Dynamics: Covalent Bonding *versus* Physical Adsorption
in Polymer Nanocomposites. ACS Nano.

[108] Karim Md. R., Vernerey F., Sain T. (2025). Constitutive Modeling
of Vitrimers and Their Nanocomposites Based on Transient Network Theory. Macromolecules.

[109] Woodruff M. A., Hutmacher D. W. (2010). The return
of a forgotten polymer—Polycaprolactone
in the 21st century. Prog. Polym. Sci..

[110] Self J. L., Dolinski N. D., Zayas M. S., Read
de Alaniz J., Bates C. M. (2018). Brønsted-Acid-Catalyzed Exchange
in Polyester Dynamic Covalent Networks. ACS
Macro Lett..

[111] Cho J., Sampaio de Almeida G.
J., Nandi M., Finn M. G., Brettmann B. (2026). Stress Relaxation Modulation via
Functionalized Silica
in Covalent Adaptable Networks. ACS Appl. Polym.
Mater..

[112] Dolinski N. D. (2026). Control of Dynamic Composites through Filler
Surface Chemistry. ACS Macro Lett..

[113] Sun J. (2023). Interfacial Compatibility
of Core–Shell Cellulose
Nanocrystals for Improving Dynamic Covalent Adaptable Networks’
Fracture Resistance in Nanohybrid Vitrimer Composites. ACS Appl. Mater. Interfaces.

[114] Anthony H., Sandra S., Yves L., Véronique M. (2026). Enhancing
reprocessability of dynamic polymer composites with thermally sensitive
particles and controlled particle-matrix interfaces. Adv. Compos. Hybrid Mater..

[115] Gaca S. (2026). Thermoset meets thermoplastic: upcycling PLA
in digital
light processing 3D printing of dynamic thiol–ene composites. Polym. Chem..

[116] Vallin A., Battegazzore D., Damonte G., Fina A., Monticelli O. (2022). On the Development
of Nanocomposite Covalent Associative
Networks Based on Polycaprolactone and Reduced Graphite Oxide. Nanomaterials.

[117] Houbben M., Sànchez C. P., Vanderbemden P., Noels L., Jérôme C. (2023). MWCNTs filled PCL covalent
adaptable networks: Towards reprocessable, self-healing and fast electrically-triggered
shape-memory composites. Polymer (Guildf)..

[118] Hong Y., Goh M. (2024). Vitrimer Nanocomposites
for Highly
Thermal Conducting Materials with Sustainability. Polymers (Basel).

[119] Benedetti L., Orikasa K., Jiménez-Suárez A., Agarwal A. (2025). Epoxy-Based Vitrimer/GNP Nanocomposites: Unleashing
Superior Shape Recovery and Multifunctional Properties. ACS Applied Engineering Materials.

[120] Arya T. (2025). “Multifunctional
vitrimer nanocomposites based
on waste plastic-derived reduced graphene oxide and epoxy/polyethylene
blends with enhanced self-healing properties,”. Polymer (Guildf).

[121] Yang Y., Pei Z., Zhang X., Tao L., Wei Y., Ji Y. (2014). Carbon nanotube–vitrimer
composite for facile
and efficient photo-welding of epoxy. Chem.
Sci..

[122] Huang J., Zhang S., Wang Q., Zhu L. (2025). Synthesis
and Induction Heating-Induced Self-Healing of Epoxy Vitrimer Nanocomposites. SSRN.

[123] Xia J. (2024). “Bio-Based Epoxy
Vitrimers with Excellent Properties
of Self-Healing, Recyclability, and Welding,”. Polymers (Basel).

[124] Huang J., Zhou J., Liu M. (2022). Interphase in Polymer
Nanocomposites. JACS Au.

[125] Zamani Zakaria A., Shelesh-Nezhad K. (2016). “Quantifying
the particle
size and interphase percolation effects on the elastic performance
of semi-crystalline nanocomposites,”. Comput. Mater. Sci..

[126] Baxter S. C., Burrows B. J., Fralick B. S. (2016). Mechanical
percolation
in nanocomposites: Microstructure and micromechanics. Probabilistic Engineering Mechanics.

[127] Kaiser S., Jandl J., Novak P., Schlögl S. (2020). Design and
characterisation of vitrimer-like elastomeric composites from HXNBR
rubber. Soft Matter.

[128] Bokobza L. (2023). Elastomer Nanocomposites: Effect
of Filler–Matrix
and Filler–Filler Interactions. Polymers
(Basel).

[129] Kumar S. K., Jouault N., Benicewicz B., Neely T. (2013). Nanocomposites with
Polymer Grafted Nanoparticles. Macromolecules.

[130] Chao H., Riggleman R. A. (2013). Effect
of particle size and grafting
density on the mechanical properties of polymer nanocomposites. Polymer (Guildf)..

[132] Wiesner B., Kohn B., Mende M., Scheler U. (2018). Polymer Chain
Mobility under Shear—A Rheo-NMR Investigation. Polymers (Basel).

[133] Xiong Y., Wu Z., Wu L., Li C., Chen W. (2024). Rheo-NMR: A versatile hyphenated technique for capturing molecular
dynamics and structure under flow. Magnetic
Resonance Letters.

[134] Karatrantos A. V., Couture O., Hesse C., Schmidt D. F. (2024). Molecular
Simulation of Covalent Adaptable Networks and Vitrimers: A Review. Polymers (Basel).

[135] Perego A., Khabaz F. (2020). “Volumetric and Rheological
Properties of Vitrimers: A Hybrid Molecular Dynamics and Monte Carlo
Simulation Study,”. Macromolecules.

[136] Duan P. (2023). Insights into Uniaxial Tension and Relaxation of Nanorod-Filled
Polymer Vitrimer Nanocomposites: A Molecular Dynamics Simulation. Macromolecules.

[137] Zheng Y., Varshney V., Vashisth A. (2025). Accelerated
ReaxFF
Simulations of Vitrimers with Dynamic Covalent Adaptive Networks. Macromolecules.

